# Demographic effects on facial emotion expression: an interdisciplinary investigation of the facial action units of happiness

**DOI:** 10.1038/s41598-021-84632-9

**Published:** 2021-03-04

**Authors:** Yingruo Fan, Jacqueline C. K. Lam, Victor O. K. Li

**Affiliations:** Department of Electrical and Electronic Engineering, The University of Hong Kong, Pokfulam, Hong Kong China

**Keywords:** Computational models, Human behaviour

## Abstract

Understanding demographic difference in facial expression of happiness has crucial implications on social communication. However, prior research on facial emotion expression has mostly focused on the effect of a single demographic factor (typically gender, race, or age), and is limited by the small image dataset collected in laboratory settings. First, we used 30,000 (4800 after pre-processing) real-world facial images from Flickr, to analyze the facial expression of happiness as indicated by the intensity level of two distinctive facial action units, the Cheek Raiser (AU6) and the Lip Corner Puller (AU12), obtained automatically via a deep learning algorithm that we developed, after training on 75,000 images. Second, we conducted a statistical analysis on the intensity level of happiness, with both the main effect and the interaction effect of three core demographic factors on AU12 and AU6. Our results show that females generally display a higher AU12 intensity than males. African Americans tend to exhibit a higher AU6 and AU12 intensity, when compared with Caucasians and Asians. The older age groups, especially the 40–69-year-old, generally display a stronger AU12 intensity than the 0–3-year-old group. Our interdisciplinary study provides a better generalization and a deeper understanding on how different gender, race and age groups express the emotion of happiness differently.

## Introduction

Human facial expressions are able to convey countless important non-verbal messages. There has been a long-standing debate over the universality of human facial emotional expression^1^ (FEE) in psychology and neuroscience. The prominent studies^2,3^ conducted by Ekman and colleagues provided a strong piece of evidence in support of the universality of FEE. Nevertheless, for over two decades, researchers have been refuting the assumption of universality, and consistently agreed on the cultural shaping of human facial expressions^4–6^. Several works^7,8^ examined the role of culture on facial expressions, in which participants were required to select facial images based on their own culture-specific intuitions and observations. Along those lines, Jack and colleagues^4^ reconstructed the dynamic mental representation of facial expressions. Their results show that the representation of emotional intensity varies across cultures. While the East Asian models express the emotional intensity primarily with the eyes, the Western Caucasian models express emotional intensity with other parts of the face. As compared to the cultural effect, the gender effect on facial expressions is more consistently reported in the literature^9–11^. A common belief is that female is more emotionally expressive than male and tends to display positive emotional states more exaggeratedly^12^, as supported in many earlier related studies^9,13,14^. Concerning the age effect on facial expressions, one early study^15^ confirmed that the face of an older person displays more mixed expressions than that of a younger person, while some other studies^16,17^ found no age effect on facial expressions. The results showing that the effect of aging vary from culture to culture may potentially be attributable to methodological disparity and small sample size.

With the prior psychological evidence concerning the difference in facial expressions and muscle activities in relation to demographic influence, this study aims to investigate the demographic effects on the facial expression of happiness (FEH) via the facial action units (FAUs) that are associated with the expression of happiness. Many studies^4–17^ examined the effect of a single demographic factor, such as gender, race, or age, on the FEE, but few explored the interaction effects of the three demographic factors on the FEE. For instance, prior study^9–14^ exploring the effect of age neglected the possibility that age might interact with gender or race to affect FEH. However, our study provides a fuller understanding of the main effect, as well as the interaction effect of race, gender and age on FEH via the FAUs of happiness. Furthermore, unlike traditional FEE data collection, which were normally conducted in controlled laboratory settings, facial expression pictures were collected under natural settings, based on an existing big and real-life facial expression database^18,19^.

As an alternative to FEE evaluation, the facial action coding system (FACS)^20^ is developed to provide a more comprehensive and objective measurement of facial expressions. FACS decomposes facial expressions into over 40 individual facial muscle movements, namely, the Action Units (AUs). A certain combination of facial AUs (FAUs) is used to denote a specific facial expression. For real-life human interaction, smiling is the most common FEH. Several studies have confirmed that the smile intensity in pictures can be used to represent the extent of marriage satisfaction^21^ and longevity^22^. Specifically, the highly popular “Duchenne smile”^23–25^ has long been considered an indicator of genuine positive emotions in social science^26^ and affective computing^27^. According to FACS, “Duchenne smile” consists of the Cheek Raiser (AU6) and the Lip Corner Puller (AU12) muscle actions. AUs are normally coded on a six-point scale (with 0 to 5 to indicate the level of emotional intensity), allowing one to quantify the demographic difference in FEH.

For the purpose of this study, we develop a deep learning-based algorithm^28^ to estimate the intensity level of two important AUs (AU6 and AU12), which are naturally associated with FEH. Our automated algorithm is based on the heatmap regression framework, which encodes the facial images to a set of heatmaps. Heatmaps can be used to represent the geometrical and spatial characteristics of specific FAUs. Besides, the heatmap pixel values can be used to reflect the FAU intensity. Recently, the popularity of online social platform, such as Flickr, offers a new source of easily and freely accessible, large-scale real-life facial image data. Analytic samples are collected from the Flickr facial image database^18^, with all facial images labeled with ‘happy’ being collected. To ensure data quality, facial images with severely occluded faces, with poor lighting, and low resolution are excluded. As such, we combined the social “big data” with our automated facial coding algorithm^28^ to examine the individual and the interactive effect of gender, age, and race on FEH. To summarize, our novelties include the following. (i) When compared with the self-reporting measures, we have created a balanced composition of facial image samples based on age, race and gender, extending beyond controlled laboratory settings to more natural settings taking natural facial images as inputs. (ii) To the best of our knowledge, this is the first study that leverages artificial intelligence (AI) techniques for measuring the FAU intensity of happiness, and analyzing the effects of three key demographic factors on FEH simultaneously. (iii) Our study demonstrates that AI-driven social “big data” analysis can be used to measure FEH, via examining the FAU intensity of happiness, to validate the theory of human emotions in three closely related fields, such as psychology, anthropology and social studies. However, FEH is a complex notion that can be influenced by various external and internal factors. Therefore, these three demographic variables are unlikely to fully capture all the differences in FEH via corresponding FAUs. Our study therefore aims to first examine how these three demographic variables would affect the intensity of FAU-associated FEH, by identifying the specific differences in FAUs of happiness across different demographic groups in details.

## Results

**Figure 1:**
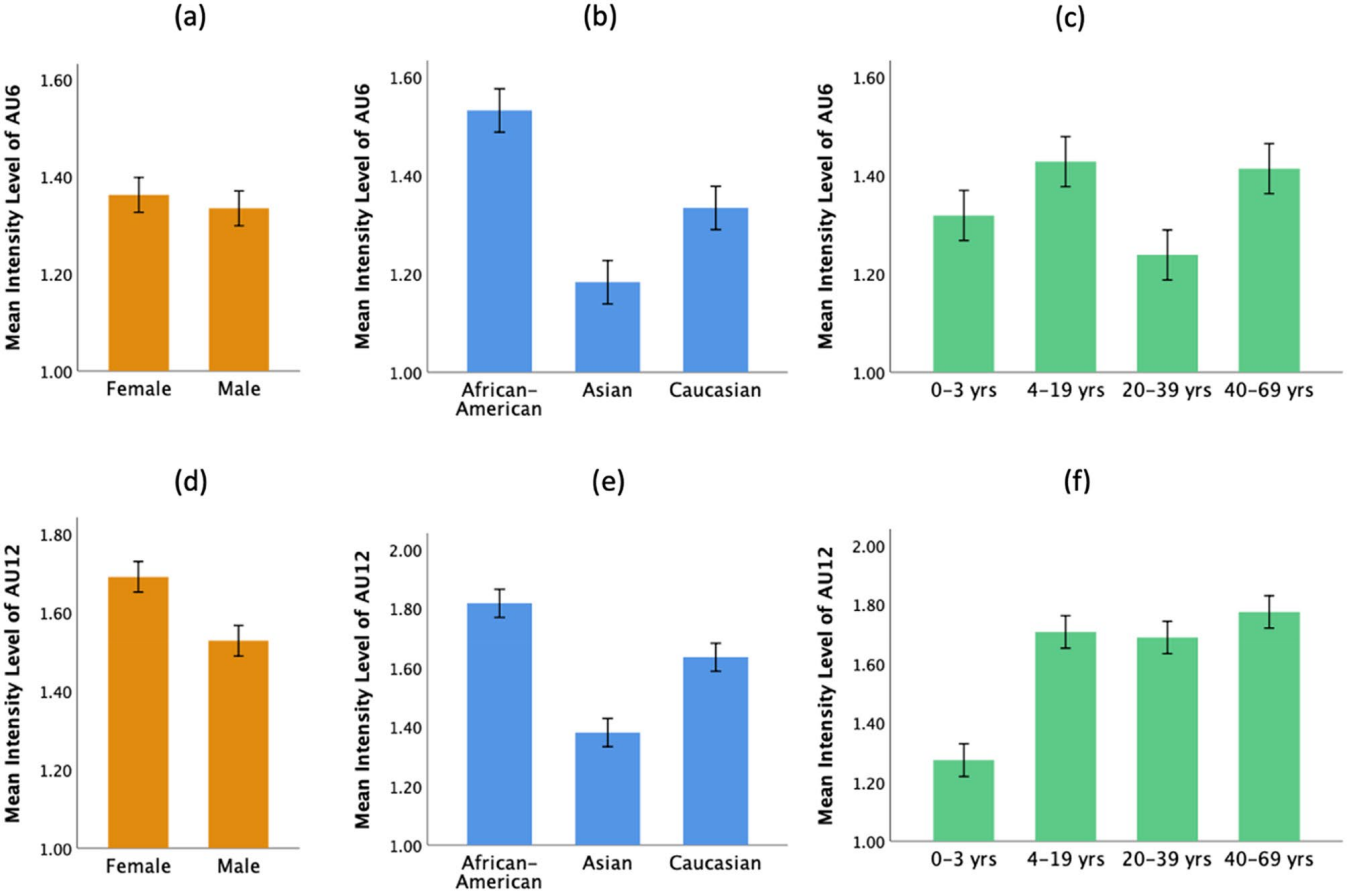
Mean AU6 intensity level and mean AU12 intensity level by gender, race and age. The error bar represents the standard error.

Figure 1 provides an overview of the three demographic variables and their effects on AU6 and AU12 intensity. The independent variables include gender (male and female), race (Caucasian, Asian and African American), and age (0–3, 4–19, 20–39, and 40–69-year-old), whereas the dependent variables are AU6 and AU12 intensity. Our results are based on a gender-, race- and age-balanced sample consisting of 4800 images, with each demographic subgroup (e.g., Caucasian, male, 0–3-year-old) consisting of 200 images. To gain a deeper understanding of the main and the interaction effect, a three-way (race × gender × age) analysis of variance (ANOVA) is conducted on AU6 and AU12 intensity separately (Table 1), followed by the posthoc comparison test that compares the means across all demographic groups. The F-value and the *p* value generated by the three-way ANOVA are used for evaluating the statistical significance; the mean difference (MD) and the corresponding standard error (SE) generated by the post-hoc comparison are used to determine if there exists any difference across groups.

**Table 1:** Three-way (Race × Gender × Age) ANOVA of the main effect and the interaction effect on (a) AU6 intensity and (b) AU12 intensity.

Factor	Sum of squares	df	Mean square	*F*-value	*p* value	Partial ${\eta }^{2}$
**(a) Three-way ANOVA on AU6 intensity**						
Race	97.476	2	48.738	63.211	< 0.001	0.026
Gender	0.921	1	0.921	1.195	0.274	< 0.001
Age	28.156	3	9.385	12.172	< 0.001	0.008
Race $\times$ Gender	7.506	2	3.753	4.868	0.008	0.002
Race $\times$ Age	18.833	6	3.139	4.071	< 0.001	0.005
Gender $\times$ Age	2.763	3	0.921	1.194	0.310	0.001
Race $\times$ Gender $\times$ Age	8.395	6	1.399	1.815	0.092	0.002
**(b) Three-way ANOVA on AU12 intensity**						
Race	153.417	2	76.709	85.101	< 0.001	0.034
Gender	31.835	1	31.835	35.318	< 0.001	0.007
Age	185.954	3	61.985	68.766	< 0.001	0.041
Race $\times$ Gender	6.573	2	3.287	3.646	0.026	0.002
Race $\times$ Age	14.796	6	2.466	2.736	0.012	0.003
Gender $\times$ Age	23.732	3	7.911	8.776	< 0.001	0.005
Race $\times$ Gender $\times$ Age	6.987	6	1.165	1.292	0.257	0.002

Significant variables and their corresponding *p* values are highlighted in grey. *p* value < 0.05 is considered as statistically significant. The effect size can be represented by the partial Eta-squared (${\eta }^{2}$), a higher value indicates a higher effect size.

The three-way ANOVA (Table 1a) reveals that race (F = 63.211, *p* < 0.001, partial ${\eta }^{2}$=0.026), age (F = 12.172, *p* < 0.001, partial ${\eta }^{2}$=0.008), race × gender interaction (F = 4.868, *p* = 0.008, partial ${\eta }^{2}$=0.002), and race × age interaction (F = 4.071, *p* < 0.001, partial ${\eta }^{2}$=0.005) have a small but significant effect on AU6 intensity. Likewise, the three-way ANOVA (Table 1b) confirms that race (F = 85.101, *p* < 0.001, partial ${\eta }^{2}$=0.034), gender (F = 35.318, *p* < 0.001, partial ${\eta }^{2}$=0.007), age (F = 68.766, *p* < 0.001, partial ${\eta }^{2}$=0.041), plus the race × gender interaction (F = 3.646, *p* = 0.026, partial ${\eta }^{2}$=0.002), race × age interaction (F = 2.736, *p* = 0.012, partial ${\eta }^{2}$=0.003), and gender × age (F = 8.776, *p* < 0.001, partial ${\eta }^{2}$=0.005) interaction have a small but significant effect on AU12.

### The main effect

#### Race main effect

The race effect on AU6 intensity and AU12 intensity is similar, as shown in Fig. 1b,e. Moreover, the race effect on AU12 intensity is stronger than that on AU6 intensity, implying that the change in the activity of muscles in the mouth area (AU12) is more pronounced than that around the cheek area (AU6). The posthoc comparison suggests that the African American group has a significantly higher AU12 intensity when compared with the Asian group (MD = 0.436, SE = 0.034, *p* < 0.001, Cohen’s d = 0.454) and the Caucasian group (MD = 0.182, SE = 0.034, *p* < 0.001, Cohen’s d = 0.183), and the Caucasian group has a significantly higher AU12 intensity when compared with the Asian group (MD = 0.254, SE = 0.034, *p* < 0.001, Cohen’s d = 0.261). A similar trend is also found in AU6 intensity. Our test results imply that the African American group and the Caucasian group will in general display a higher intensity of AU6 and AU12 than the Asian group.

A cross-cultural psychological study^29^ revealed that cultural difference in the arousal emotional expression between the East and the West is prominent. People in the West tend to embrace a higher arousal emotional state, whilst people in the East tend to embrace a lower arousal emotional state^29,30^. Besides, the cultural theory^31^ posited that the Chinese culture and the American culture conceptualize happiness differently. For Americans, happiness is conceived as being upbeat and unmistakably positive, whilst Chinese tend to conceive happiness as a more solemn and calm entity^31^. Such difference in the conceptualization of happiness between the West and the East might explain why the Caucasian group and the Asian group display highly different AU6 and AU12 intensity.

#### Gender main effect

Based on the three-way ANOVA, a significant gender effect on AU12 intensity (F = 35.318, *p* < 0.001, partial ${\eta }^{2}$=0.007) has been identified, whilst no significant gender effect on AU6 intensity (F = 1.195, *p* = 0.274, partial ${\eta }^{2}$<0.001) has been found. Accordingly, the posthoc test suggests that the female group shows a significantly higher AU12 intensity as compared to the male group (MD = 0.163, SE = 0.027, *p* < 0.001, Cohen’s d = 0.165). The significant difference in AU12 intensity can also be observed in Fig. 1d, indicating that the female group is generally more expressive in happiness, giving a higher AU12 intensity (or a bigger smile) than the male group.

From a psychological perspective. Dodd et al.^32^ concluded that gender difference can be detected in the way people smile and is linked to the cultural expectation that females should behave friendlier and are more emotionally expressive than males. Interestingly, Clancy and Dollinger^33^ provided a compelling evidence that such gender difference in smiling can be attributable to females’ greater tendency to seek social connectedness than males. Besides, various studies^11,34–36^ supported the view that females are more expressive than males. This experimental and statistical study will partially support these psychological views on emotional expressiveness variation by gender.

#### Age main effect

The three-way ANOVA result on AU6 and AU12 intensity (Table 1) indicates that age has a small but significant effect on both FAUs (AU6: F = 12.172, *p* < 0.001, partial ${\eta }^{2}$=0.008; AU12: F = 68.766, *p* < 0.001, partial ${\eta }^{2}$=0.041). In addition, the posthoc comparison finds that the mean AU6 intensity of the 40–69-year-old group is significantly higher than that of the 20–39-year-old (MD = 0.197, SE = 0.036, *p* < 0.001, Cohen’s d = 0.191). Previous finding^37^ suggested that wrinkles, folds and lower expressivities of older faces may affect how facial expressions are being decoded. Based on this observation, we hypothesize that these facial features due to old age may also affect the facial expression of happiness, resulting in a lower AU6 intensity across the old age group. Concerning the age-related effect on facial expressions, several studies^16,17,38^ found no difference in expressivity across the younger and the older age groups. These results contradict our newly generated results supporting that the older age group may express happiness more intensively than the younger age group. It would be worthwhile to conduct more studies to investigate the underlying mechanisms that govern the facial expressions of different age groups.

**Figure 2:**
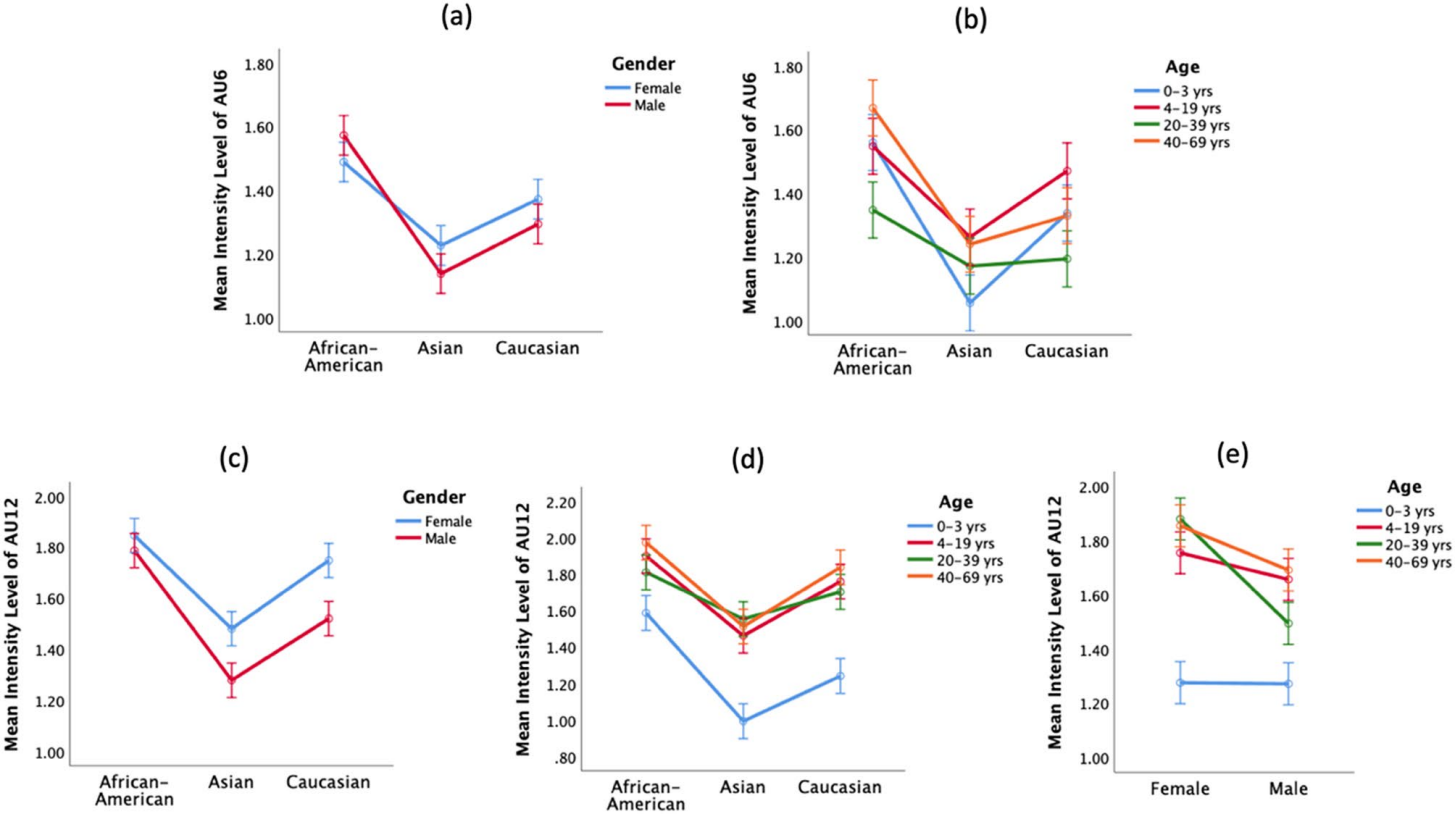
Statistically significant two-way interactions on AU6 and AU12 intensity (*p* < 0.05): (**a**) race × gender interaction on AU6 intensity, (**b**) race × age interaction on AU6 intensity, (**c**) race × gender interaction on AU12 intensity, (**d**) race × age interaction on AU12 intensity, and (**e**) gender × age interaction on AU12 intensity. The error bar represents the standard error.

Our results show that the 4–19-year-old, the 20–39-year-old and the 40–69-year-old have a significantly higher AU12 intensity than the 0–3-year-old (4–19-year-old: MD = 0.432, SE = 0.039, *p* < 0.001, Cohen’s d = 0.467; 20–39-year-old: MD = 0.414, SE = 0.039, *p* < 0.001, Cohen’s d = 0.451; 40–69-year-old: MD = 0.500, SE = 0.039, *p* < 0.001, Cohen’s d = 0.553). Figure 1f clearly shows there is an obvious big mean difference between the 0–3-year-old and the other age groups, indicating that the infant group has a lower AU12 intensity than the rest of the age groups. With respect to the race × age interaction effect (Fig. 2d), across all race groups, there is a big mean difference between the 0–3-year-old and the other age groups. Hence, the big mean difference is not caused by a specific race group. Amongst all age groups, the 4–19-year-old smile much more intensively via AU12 when compared to the 0–3-year-old infant group.

### The interaction effect

#### Race × Gender interaction effect on AU6 intensity

As displayed in Table 1a, the race × gender interaction effect on AU6 intensity is small but significant (F = 4.868, *p* = 0.008, partial ${\eta }^{2}$=0.002), indicating that there is a statistically significant difference in the expression of AU6 intensity across the female group and the male group when race is being controlled for (see also Fig. 2a). To reveal whether certain race group shows a higher AU6 intensity on the female group than on the male group, we further analyzed the gender effect on each of the three race groups by the posthoc comparison. Our result shows that the Asian female group exhibits a marginally higher AU6 intensity than the Asian male group (MD = 0.089, SE = 0.044, *p* = 0.043, Cohen’s d = 0.100). On the contrary, the African American group and the Caucasian group do not show any significant difference between females and males.

We then analyzed the gender effect on race. The univariate test shows that different races have displayed a significant statistical difference on AU6 intensity across both the female group (F = 17.816, *p* < 0.001), and the male group (F = 38.754, *p* < 0.001). For the female group, the African-American group exhibits a significantly higher AU6 intensity when compared with the Asian group (MD = 0.262, SE = 0.044, *p* < 0.001, Cohen’s d = 0.451) and the Caucasian group (MD = 0.116, SE = 0.044, *p* = 0.024, Cohen’s d = 0.451), while the Caucasian group has displayed a significantly higher AU6 intensity when compared with the Asian group (MD = 0.145, SE = 0.044, *p* = 0.003, Cohen’s d = 0.451). A similar trend is found among the male group, as observed in (Fig. 2a).

#### Race × Age interaction effect on AU6 intensity

As shown in Fig. 2b, there is a small but statistically significant race × age interaction effect on AU6 intensity (F = 4.071, *p* < 0.001, partial ${\eta }^{2}$=0.005), implying that different age groups have displayed a statistically significant difference in the expression of AU6 intensity, when race is being controlled for. To reveal whether certain race group displays a higher AU6 intensity on one age group than on the others, we further analyze the age effect on each of the three race groups by the posthoc comparison.

For the African American cohort, the 40–69-year-old exhibits a significantly higher AU6 intensity than the 20–39-year-old (MD = 0.320, SE = 0.062, *p* < 0.001, Cohen’s d = 0.350). For the Asian, the 4–19-year-old and the 40–69-year-old display a statistically significantly higher AU6 intensity than the 0–3-year-old (4–19-year-old: MD = 0.206, SE = 0.062, *p* = 0.005, Cohen’s d = 0.240; 40–69-year-old: MD = 0.184, SE = 0.062, *p* < 0.05, Cohen’s d = 0.206). For the Caucasian cohort, the 4–19-year-old turns out to have the highest AU6 intensity across all age groups, especially showing a significantly higher AU6 intensity than the 20–39-year-old (MD = 0.276, SE = 0.062, *p* < 0.001, Cohen’s d = 0.314). However, for the Caucasian and the Asian cohort, the 40–69-year-old and the 20–39-year-old do not show any significant difference in AU6 intensity (Caucasian: MD = 0.135, SE = 0.062, *p* > 0.1, Cohen’s d = 0.152; Asian: MD = 0.069, SE = 0.062, *p* > 0.001, Cohen’s d = 0.076).

#### Race × Gender interaction effect on AU12 intensity

To decompose the race × gender interaction on AU12 intensity, we analyzed the race effect on AU12 intensity after controlling for gender, as well as the gender effect on AU12 intensity after controlling for race. For both the female group and the male group, the African American group displays a much higher AU12 intensity than that of the Caucasian group (Female: MD = 0.098, SE = 0.047, *p* = 0.118, Cohen’s d = 0.100; Male: MD = 0.266, SE = 0.047, *p* < 0.001, Cohen’s d = 0.265) and the Asian group (Female: MD = 0.365, SE = 0.047, *p* < 0.001, Cohen’s d = 0.372; Male: MD = 0.507, SE = 0.047, *p* < 0.001, Cohen’s d = 0.543). Further, the posthoc comparison reveals that the gender difference in AU12 intensity is more pronounced for the Caucasian group. Specifically, the female group exhibits a significantly higher AU12 intensity as compared to the male group for both the Caucasian group (MD = 0.228, SE = 0.047, *p* < 0.001, Cohen’s d = 0.227) and the Asian group (MD = 0.202, SE = 0.047, *p* < 0.001, Cohen’s d = 0.217), but no significant gender difference in the display of AU12 intensity is identified for the African American group.

#### Race × Age interaction effect on AU12 intensity

To decompose the race × age interaction effect on AU12 intensity, we first analyzed the race effect on AU12 intensity after controlling for age, then the age effect on AU12 intensity after controlling for race. Table 2 summarizes the results. Across all age groups, the African American group’s mean AU12 intensity is significantly higher than that of the Asian group (see Fig. 2d and Table 2a). Across all race groups, the 4–19-year-old, the 20–39-year-old and the 40–69-year-old exhibit a significantly higher AU12 intensity than the 0–3-year-old, as shown in Fig. 2d and Table 2b. For the African American cohort, the 40–69-year-old smiles more intensively in AU12 than the rest of the age groups, but the difference is not statistically significant when compared with the 4–19-year-old. For the Asian cohort, the young adult group (20–39-year-old) displays a bigger smile intensity in AU12 than the rest of the age groups, but the difference is not statistically significant, except when compared with the 0–3-year-old. For the Caucasian cohort, the old age group (40–69-year-old) displays a bigger smile intensity in AU12 than the rest of the age groups, but the difference is not statistically significant when compared with the 4–19-year-old.

**Table 2:** Pairwise comparison of the Race × Age interaction effect on AU12 intensity.

Age	(I) Race	(J) Race	*MD* (I–J)	*SE*	*p* value	Cohen’s d
**(a) Race effect on AU12 intensity after controlling for Age**						
0–3	African-American	Asian	0.592	0.067	< 0.001	0.868
	African-American	Caucasian	0.345	0.067	< 0.001	0.428
	Caucasian	Asian	0.247	0.067	0.001	0.325
	African-American	Asian	0.436	0.067	< 0.001	0.427
4–19	African-American	Caucasian	0.140	0.067	0.111	0.134
	Caucasian	Asian	0.296	0.067	< 0.001	0.289
	African-American	Asian	0.256	0.067	< 0.001	0.246
20–39	African-American	Caucasian	0.107	0.067	0.335	0.103
	Caucasian	Asian	0.149	0.067	0.079	0.148
	African-American	Asian	0.460	0.067	< 0.001	0.468
40–69	African-American	Caucasian	0.136	0.067	0.127	0.135
	Caucasian	Asian	0.323	0.067	< 0.001	0.333
**(b) Age effect on AU12 intensity after controlling for Race**						
African-American	0–3	4–19	− 0.312	0.067	< 0.001	0.346
	0–3	20–39	− 0.222	0.067	0.006	0.243
	0–3	40–69	− 0.386	0.067	< 0.001	0.434
	4–19	20–39	0.090	0.067	0.182	0.085
	4–19	40–69	− 0.074	0.067	0.270	0.072
	20–39	40–69	− 0.164	0.067	0.015	0.157
Asian	0–3	4–19	− 0.468	0.067	< 0.001	0.561
	0–3	20–39	− 0.558	0.067	< 0.001	0.663
	0–3	40–69	− 0.518	0.067	< 0.001	0.649
	4–19	20–39	− 0.091	0.067	0.176	0.090
	4–19	40–69	− 0.051	0.067	0.450	0.052
	20–39	40–69	0.040	0.067	0.551	0.041
Caucasian	0–3	4–19	− 0.517	0.067	< 0.001	0.536
	0–3	20–39	− 0.461	0.067	< 0.001	0.491
	0–3	40–69	− 0.595	0.067	< 0.001	0.634
	4–19	20–39	0.056	0.067	0.402	0.055
	4–19	40–69	− 0.078	0.067	0.247	0.076
	20–39	40–69	− 0.134	0.067	0.046	0.134

(a) Race effect on AU12 intensity after controlling for Age; (b) Age effect on AU12 intensity after controlling for Race. Statistically significant mean difference (MD) is highlighted in grey. *p* value < 0.05 is considered statistically significant. The effect size is determined by Cohen’s d. A higher value indicates a larger standardized mean difference between groups.

#### Gender × Age interaction effect on AU12 intensity

Lastly, we analyzed the gender × age interaction effect on AU12 intensity. We first analyzed the age effect on AU12 intensity after controlling for gender, then the gender effect on AU12 intensity after controlling for age. For the age effect on AU12 intensity, for the female group, the 20–39-year-old, the 40–69-year-old, and the 4–19-year-old, have a significantly higher AU12 intensity as compared to the 0–3-year-old (20–39-year-old: MD = 0.605, SE = 0.055, *p* < 0.001, Cohen’s d = 0.676; 40–69-year-old: MD = 0.579, SE = 0.055, *p* < 0.001, Cohen’s d = 0.631; 4–19-year-old: MD = 0.479, SE = 0.055, *p* < 0.001, Cohen’s d = 0.520); while the 20–39-year-old female group tends to have a slightly higher AU12 intensity as compared to the 40–69-year-old female group, even though the difference is not statistically significant (MD = 0.025, SE = 0.055, *p* > 0.001, Cohen’s d = 0.025). For the male group, the 40–69-year-old tends to display the highest AU12 intensity as compared to the other ages, and a much higher AU12 intensity is displayed by the 40–69-year-old male group than the 20–39-year-old male group (MD = 0.197, SE = 0.055, *p* < 0.05, Cohen’s d = 0.197). We studied the gender effect on AU12 intensity after controlling for age; for the 20–39-year-old group, there is a statistically significant gender effect on AU12 intensity with the female group having a significantly higher AU12 intensity than the male group (MD = 0.386, SE = 0.055, *p* < 0.001, Cohen’s d = 0.381); the same pattern is also found across the 40–69-year-old group (MD = 0.164, SE = 0.055, *p* = 0.003, Cohen’s d = 0.163). However, there is no statistically significant gender difference in AU12 intensity between the 0–3-year-old group and the 4–19-year-old group (see Fig. 2e).

Figure 2c–e summarize the Race × Gender interaction (F = 3.646, *p* = 0.026, partial ${\eta }^{2}$=0.002), race × age interaction (F = 2.736, *p* = 0.012, partial ${\eta }^{2}$=0.003), and Gender × Age interaction (F = 8.776, *p* < 0.001, partial ${\eta }^{2}$=0.005) on AU12 intensity, respectively.

## Discussion

The demographic effect on FEE has been studied across different disciplines, with most of the research focusing on a single demographic factor. In this study, we examined the main and the interaction effect of three demographic factors on FEH via studying the FAUs of happiness. Meanwhile, we acknowledge the ongoing debates about the relationship between human expressions and emotions^39^.

Instead of basing on self-reporting information, we adopted a more objective and standardized approach for measuring AU intensities, which may suffer less human perception bias. The analysis of the main effect on both AU6 and AU12 intensity suggests that, first, in general, the female group shows a higher AU12 intensity than the male group in FEH. The result agrees with the traditional view^12^ that the female group is more emotionally expressive than the male group and is more likely to show their positive emotional state of happiness more expressively. Our result is consistent with the previous findings^11,34–36^. Second, when comparing the three different races, including African American, Caucasian and Asian, the African American group tends to have a higher AU6 and AU12 intensity in FEH than the other two groups, while the Caucasian group has a higher AU6 and AU12 intensity than the Asian group. Although the race effect may vary somehow by gender or age, the overall trend of the average AU6 and AU12 intensity is consistent, as revealed in Fig. 1b,e.

Third, our results show that the race effect interacts with the age effect to affect AU6 and AU12 intensity, respectively, as shown in Fig. 2b,d. In terms of AU6 intensity, for the African American cohort, the 40–69-year-old exhibits a significantly higher intensity than the 20–39-year-old. For the Asian cohort, the 4–19-year-old and the 40–69-year-old display a significantly higher AU6 intensity than the 0–3-year-old. For the Caucasian cohort, the 4–19-year-old has the highest AU6 intensity across all age groups, especially showing a significantly higher AU6 intensity than the 20–39-year-old. However, for the Caucasian and the Asian cohort, the 40–69-year-old and the 20–39-year-old do not show any significant difference in AU6 intensity and the difference is very small. In terms of AU12 intensity, for the African American cohort, the 40–69-year-old age smiles more intensively than the rest of the age groups, but the difference is very small and not statistically significant when compared with the 4–19-year-old. For the Asian cohort, the young adult group (20–39-year-old) displays a bigger AU12 intensity than the rest of the age groups, but the difference is very small and not statistically significant, except when compared with the 0–3-year-old. For the Caucasian cohort, the old age group (40–69-year-old) displays a bigger AU12 intensity than the rest of the age groups, but the difference is very small and not statistically significant when compared with the 4–19-year-old.

Fourth, significant age-related difference in AU6 and AU12 intensity can also be identified. To our surprise, the 4–19-year-old, the 20–39-year-old and the 40–69-year-old have an average AU12 intensity significantly higher than that of the 0–3-year-old (see Fig. 1f). Lastly, the gender difference has a more pronounced effect on the 20–39-year-old group’s AU12 intensity as compared to the 0–3-year-old group (Fig. 1e). In particular, the female exhibits a significantly higher AU12 intensity as compared to the male across the 20–39-year-old, whereas no significant gender difference in AU12 intensity is observed for the 0–3-year-old. What might be the reason that the gender difference in AU12 intensity is not obvious in the early (0–3) years? We speculate that this may be partially attributable to the lesser muscle activities in the mouth area during infancy^37^.

In the big data era, social network platforms have been extensively utilized for emotion^40^ or personality^41^ analysis. Our study has combined social “big data” in conjunction with FAU recognition technologies to address our social science/psychological research question. However, social big data have presented some limitations and call for further innovations. Despite Flickr’s popularity, our current Flickr sample may not be fully representative of the entire American population. Studies^42–45^ show that data from the social media may not be necessarily representative of the entire population. However, with the introduction of our data balance techniques, we have managed to improve the representativeness of our sample based on the facial images downloadable from Flickr. We acknowledge that human facial expressions are complex and can be influenced by various external and internal factors, types of expressions (posed vs. spontaneous), cultural backgrounds, where the expressions have been made and how the pictures of these expressions have been taken (selfies or pictures taken by others). Hence, demographic factors such as age, gender and race may not fully explain how facial expressions differ across different people. However, even if the three demographic factors may exhibit small differences in terms of the expression of happiness (with small effect sizes), they may still carry a significant statistical effect. Further, small effects can have large aggregated consequences^46^. Currently, our collected Flickr images cover both posed and spontaneous facial expressions. Hence, no distinction regarding the naturalness of our facial expressions (whether they are posed or spontaneous) can be made in our study. Our conclusions on the effects of race, age, and gender on FAUs of happiness are drawn with this limitation in mind. In the future, we will take into account the naturalness of the facial image expressions in our model, in order to obtain a more rigorous understanding of the demographic effects on FAUs of happiness.

As compared to other existing social science or psychologically driven facial-recognition studies, our study is superior in three dimensions. First, we have created a balanced composition of facial image samples based on age, race and gender, extending beyond controlled laboratory settings to more natural settings taking the natural facial images as the inputs. Second, utilizing automated AI-driven techniques for measuring the FAU intensity of happiness, we analyzed the effects of three key demographic factors on FEH simultaneously (studying both their single and interactive effects on the FAU of happiness and FEH). Third, our results have demonstrated that AI-driven social “big data” analysis can be used to measure FEH, via examining the FAU intensity of happiness, to validate theories of human emotions in three closely related fields, including psychology, anthropology and social studies.

## Conclusion

In summary, the present study investigates the statistical effects of three demographic factors, namely, gender, race and age, on the FAUs of happiness using human facial images from Flickr and an automated AI-driven FAU recognition algorithm. Specifically, two important FAUs associated with happiness are used as the dependent variables in our statistical analysis. Our method uses the FAU recognition algorithm developed by us and the natural facial image data downloaded from Flickr to provide a full understanding of the main and the interaction effects of gender, race, and age on the FAUs of happiness. Given the strong link between the FAUs of happiness and FEH, our study provides new insights into how the FAU intensity of happiness and FEH can vary across different demographic groups. Moreover, our FAU recognition and social media-based image collection methodology can provide a completely new avenue for decoding human FEE and facilitating future FEE studies on happiness and well-being. To further improve our research design, more image data can be added to each demographic group, while additional demographic factors that potentially affect FEH or other emotions can be considered in future FAU-based FEE studies.

In our statistical analysis, both the main and the interaction effect of three core demographic factors on AU12 and AU6 have been thoroughly examined. Based on Flickr dataset, our results have suggested that females are generally having a higher AU12 intensity than males. African Americans are having a higher AU6 and AU12 intensity, when compared with Caucasians and Asians. In addition, the older age groups, especially the 40–69-year-old, are generally displaying a stronger AU12 intensity than the 0–3-year-old. Hence, our interdisciplinary study provides a more automatic and a better generalization, as well as a deeper understanding on how different gender, race and age groups of the Flickr population express their emotion of happiness differently, partially representing the FEH of the Americans.

## Method

**Figure 3:**
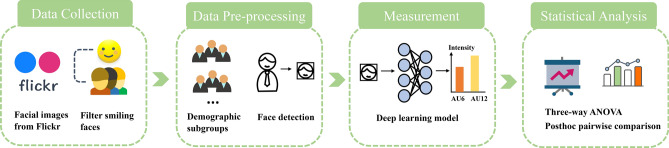
Overarching methodology.

Figure 3 summarizes our overarching methodology. We started with data collection and pre-processing. Next, we obtained the intensity level of AU6 and AU12 for three demographic groups and their interacting sub-groups using our FAU deep learning algorithm. Finally, we performed statistical analysis to investigate the single and interaction demographic effects on AU6 and AU12 intensity.

### Data collection

We collected facial images from the existing large-scale Real-world Affective Faces Database^18,19^ (RAF-DB), which is publicly available and widely used by the research community. RAF-DB contains about 30,000 real-world facial expression images downloadable from Flickr, partially representative of the facial images of the American population. As stated in the database description^18^, the images with facial expressions of happiness were retrieved from Flickr using keyword search, based on a set of emotion keywords, such as “happy” and “smile”, etc. Besides, 315 human annotators were asked to label the images with metadata including gender, race, age-range and emotion categories. Flickr is a well-established database that provides facial expressions in the natural settings. The facial expressions of people across a wide age range create a new avenue for us to examine how race or gender effect on the FAUs of happiness and FEH vary across the age range. Previous research studies usually do not consider fully how the age factor will affect AU6 and AU12 intensity after controlling for race or gender. In addition, our study refines the categorization of age groups, for instance, we have divided the 0–19-year-old age group into the 0–3-year-old and the 4–19-year-old one. This allows us to examine the difference in FAU intensity across different age groups in finer granularity. We selected any images labelled with “happy” and with a full facial profile in the original RAF-DB database to our own database. Any severely occluded faces having poor lighting and low resolution were removed. This resulted in 5585 images. Second, we separated the images into 24 subgroups based on the demographic categories, i.e., race (Caucasian, Asian, and African American), gender (male and female), and age (0–3, 4–19, 20–39, and 40–69-year-old). Third, we additionally augmented the subgroups “African-American, female, 0–3-year-old”, “Asian, female, 0–3-year-old”, and “Asian, female, 0–3-year-old” with pictures from Flickr, to ensure that the number of pictures is distributed relatively evenly across all subgroups. In addition, random down-sampling and augmentation have been utilized to ensure that our sample consists of evenly distributed demographic sub-groups. Finally, we obtained a database with 4800 images.

### Data pre-processing

During the data pre-processing stage, first, we utilized an open-source C++ library Dlib^47^ to detect and extract 68 facial landmarks, i.e., the (x, y)-coordinates of 68 facial key points in the image. Second, based on the 68 facial landmarks, we applied affine transformations (scaling, rotating, translating, etc.) to project the image into a new output coordinate space, where the positions and sizes of all human faces were approximately uniform. Third, the detected face regions were cropped and resized to a resolution of 256 × 256 pixels, which is the standard input size of our developed AU intensity estimator^28^. Given the unequal distribution of the subgroups, we performed random down-sampling for the subgroups with a large number of images, with data augmentation performed for any subgroups that have very few images. Data augmentation was implemented by randomly rotating images by − 10° to + 10° with Gaussian noises of different variances added (0.001–0.02). This step ensured that an evenly distributed sample size can be achieved across all subgroups. Finally, we obtained 200 facial expression images for each demographic subgroup, leading to a gender-, race- and age-balanced sample consisting of 4800 images for our experimental study and data analysis.

### Measurement

**Figure 4:**
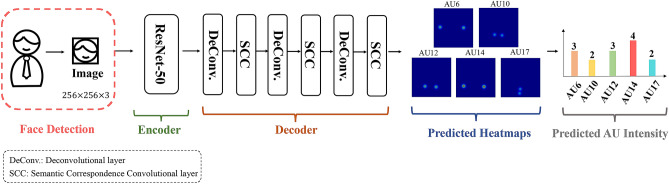
AU intensity estimation model. Given the input images, the output is a set of heatmaps for inferring the intensity level of each AU.

In this study, the dependent variables are AU6 and AU12 intensity, the two core indicators for FEH. To estimate the corresponding AU intensity of a facial image, we have developed a heatmap regression framework^28^ based on Convolutional Neural Networks (CNNs). Figure 4 illustrates the structure of our proposed heatmap regression framework. It is an Encoder-Decoder architecture, where the encoder is ResNet-50^﻿48﻿^ and the decoder consists of three deconvolutional layers and three semantic correspondence convolutional layers^28^. The deep learning models are developed based on Tensorflow^﻿49^, and the training is conducted on a server configured with eight NVIDIA GeForce GTX 1080Ti 11G GPUs. The trained deep learning model is validated using a sample of approximately 70,000 facial images from a challenging benchmark dataset^﻿50﻿^. Our automated algorithm has achieved a superior performance for the estimation of the intensity of spontaneous FAUs of happiness, as demonstrated in our previous study^28^. During the inference stage, the output provides estimates for the intensity of five fundamental AUs of happiness, including AU6 and AU12 intensity. We applied this model to calculate the intensity level of AU6 and AU12 for each demographic subgroup. Details of the FAU estimation model can be found in the Supplementary Material.

### Statistical analysis

All statistical analyses were performed using IBM SPSS version 26.0.0. Three-way analyses of variance (ANOVAs) were carried out to examine the effects of three independent demographic variables (gender, race, and age) on the dependent variables (AU6 and AU12 intensity). Follow-up simple effect analyses were conducted when the interaction effects are statistically significant. Besides, to compare the mean AU6 and AU12 intensity of different demographic subgroups, we used the posthoc pairwise multiple comparison test, with Bonferroni correction; the α level for all analyses was set at 0.05. The validity of the result can be tested by the mean difference (MD) and the corresponding standard error (SE). *p* value < 0.05 is considered as statistically significant.

## Supplementary Information

Supplementary Information.

## Data Availability

The database that supports the findings of our study is publicly available and can be requested from http://www.whdeng.cn/RAF/model1.html. The previous publications^18,19^ provide more details of the dataset.
