# Supplementary material for: Demographic effects on facial emotion expression: an interdisciplinary investigation of the facial action units of happiness
Source: Sci Rep. 2021 Mar 4;11:5214. doi: 10.1038/s41598-021-84632-9 (PMC7970860; doi:10.1038/s41598-021-84632-9)
Supplement: Supplementary file 1 — Supplementary Information. [file 41598_2021_84632_MOESM1_ESM.docx]

**Supplementary Material for Demographic Effects on Facial Emotion Expression: An Interdisciplinary Investigation of the Facial Action Units of Happiness**

**Yingruo Fan^1,†^, Jacqueline CK Lam^1,2, †,*^ and Victor OK Li^1,*^**

^1^Department of Electrical and Electronic Engineering, The University of Hong Kong, Pokfulam, Hong Kong, China

^2^Department of Computer Science and Technology, The University of Cambridge, Cambridge, United Kingdom

**^†^**both authors have equal contributions

*corresponding authors (jcklam@eee.hku.hk, vli@eee.hku.hk)

In the main manuscript, we use the algorithm [1] as a tool to estimate the intensity of FAU6 and FAU12. Here we shall explain the method in further details. In Facial Action Coding System (FACS), FAU6 denotes the activity of Cheek Raiser that appears in the cheek region. Similarly, FAU12 denotes the activity of Lip Corner Puller that appears in the mouth corner region. Therefore, we predefine the central location of each FAU according to the region where it appears.

In our method [1], we define the FAU locations using the 68 facial landmarks (FL), as shown in Figure S1. We show the predefined FAU locations in Figure S2 and present the formula w.r.t. the connection between FAU locations and the FLs in Table S1. Please note that the intensity of FAU6 and FAU12 are not measured using the calculated distance between the landmarks. We only use the facial landmarks to calculate the locations of two FAUs, as shown in Table S1. For example, the location of FAU12 is defined using the coordinates of the 49^th^ facial landmark and the 55^th^ facial landmark, whereas for the location of FAU6, the 3^rd^, 42^nd^, 47^th^, and 15^th^ facial landmarks are used.

After the FAU locations have been defined, the algorithm is implemented based on the heatmap regression framework, which encodes the facial images to a set of heatmaps. The intensity of FAU6 and FAU12 are then measured using the heatmap pixel values.


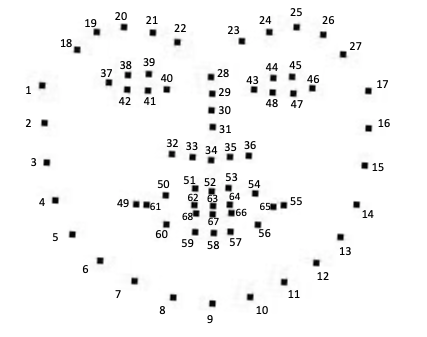


Figure S1: The 68 facial landmarks (FL).


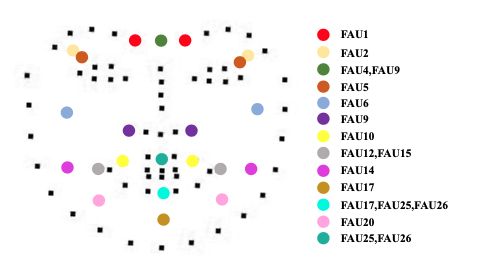


Figure S2: Predefined FAU locations.

Table S1: Formula showing the relationship between the FAU locations and the facial landmarks (FL).

| FAU | Locations (X,Y) |
| --- | --- |
| FAU6 | $(\frac{{FL}_{3}\left( x,y \right)+{FL}_{42}\left( x,y \right)}{2}$, $\frac{{FL}_{47}\left( x,y \right)+{FL}_{15}\left( x,y \right)}{2})$ |
| FAU12 | $({FL}_{49}\left( x,y \right),{FL}_{55}\left( x,y \right))$ |

To test the validity of our algorithm [1], we used Intra-Class Correlation (ICC) scores. For FAU intensity estimation, we compared the performance of our algorithm with other established algorithms on the development set of BP4D [8] in Table S2. As observed from the table, our approach gives comparable or better results than the state-of-the-art approaches listed below.

Table S2: ICC Comparison with other algorithms on the BP4D dataset.

| Algorithms | FAU6 | FAU10 | FAU12 | FAU14 | FAU17 | Avg. ICC |
| --- | --- | --- | --- | --- | --- | --- |
| BORMIR [2] | 0.73 | 0.68 | 0.86 | 0.37 | 0.47 | 0.62 |
| CCNN-IT [3] | 0.75 | 0.69 | 0.86 | 0.40 | 0.45 | 0.63 |
| KBSS [4] | 0.76 | 0.75 | 0.85 | 0.49 | 0.51 | 0.67 |
| TL [5] | 0.79 | 0.76 | 0.84 | 0.52 | 0.67 | 0.72 |
| ABSS [6] | 0.76 | 0.74 | 0.85 | 0.54 | 0.53 | 0.69 |
| ResNet-Deconv [1] | 0.70 | 0.77 | 0.78 | 0.59 | 0.49 | 0.67 |
| Hourglass [7] | 0.63 | 0.70 | 0.74 | 0.52 | 0.38 | 0.59 |
| Ours [1] | 0.74 | 0.82 | 0.86 | 0.68 | 0.51 | 0.72 |

**Reference**

[1] Yingruo Fan, Jacqueline Lam, and Victor Li. "Facial Action Unit Intensity Estimation via Semantic Correspondence Learning with Dynamic Graph Convolution." *Proceedings of the* *AAAI Conference on Artificial Intelligence*. 2020.

[2] Zhang, Yong, et al. "Bilateral ordinal relevance multi-instance regression for facial action unit intensity estimation." *Proceedings of the IEEE Conference on Computer Vision and Pattern Recognition*. 2018.

[3] Walecki, Robert, et al. "Deep structured learning for facial action unit intensity estimation." *Proceedings of the IEEE Conference on Computer Vision and Pattern Recognition*. 2017.

[4] Zhang, Yong, et al. "Weakly-supervised deep convolutional neural network learning for facial action unit intensity estimation." *Proceedings of the IEEE Conference on Computer Vision and Pattern Recognition*. 2018.

[5] Ntinou, Ioanna, et al. "A Transfer Learning approach to Heatmap Regression for Action Unit intensity estimation." *arXiv preprint arXiv:2004.06657.* 2020.

[6] Mohammadi, Mohammad R., Emad Fatemizadeh, and Mohammad H. Mahoor. "An adaptive Bayesian source separation method for intensity estimation of facial AUs." *IEEE Transactions on Affective Computing* 10.2 (2017): 144-154.

[7] Newell, Alejandro, Kaiyu Yang, and Jia Deng. "Stacked hourglass networks for human pose estimation." *European Conference on Computer Vision*. Springer, Cham, 2016.

[8] Zhang, Xing, et al. "Bp4d-spontaneous: a high-resolution spontaneous 3d dynamic facial expression database." *Image and Vision Computing* 32.10 (2014): 692-706.
